# The Drosophila melanogaster PeptideAtlas facilitates the use of peptide data for improved fly proteomics and genome annotation

**DOI:** 10.1186/1471-2105-10-59

**Published:** 2009-02-11

**Authors:** Sandra N Loevenich, Erich Brunner, Nichole L King, Eric W Deutsch, Stephen E Stein, The FlyBase Consortium, Ruedi Aebersold, Ernst Hafen

**Affiliations:** 1 Institute of Molecular Systems Biology, ETH Zurich, 8093 Zurich, Switzerland; 2 Center for Model Organism Proteomes, University of Zurich, 8057 Zurich, Switzerland; 3 Ph.D. Program in Molecular Life Sciences, University of Zurich, 8093 Zurich, Switzerland; 4 Institute for Systems Biology, Seattle, WA 98103-8904, USA; 5 Incorporated Research Institutions for Seismology, Data Management Center, Seattle, WA 98105, USA; 6 National Institute of Standards & Technology, Gaithersburg, MD 20899-8380, USA; 7 Faculty of Science, University of Zurich, 8057 Zurich, Switzerland; 8 Center for Systems Physiology and Metabolic Diseases, ETH Zurich, 8093 Zurich, Switzerland

## Abstract

**Background:**

Crucial foundations of any quantitative systems biology experiment are correct genome and proteome annotations. Protein databases compiled from high quality empirical protein identifications that are in turn based on correct gene models increase the correctness, sensitivity, and quantitative accuracy of systems biology genome-scale experiments.

**Results:**

In this manuscript, we present the *Drosophila melanogaster *PeptideAtlas, a fly proteomics and genomics resource of unsurpassed depth. Based on peptide mass spectrometry data collected in our laboratory the portal  allows querying fly protein data observed with respect to gene model confirmation and splice site verification as well as for the identification of proteotypic peptides suited for targeted proteomics studies. Additionally, the database provides consensus mass spectra for observed peptides along with qualitative and quantitative information about the number of observations of a particular peptide and the sample(s) in which it was observed.

**Conclusion:**

PeptideAtlas is an open access database for the *Drosophila *community that has several features and applications that support (1) reduction of the complexity inherently associated with performing targeted proteomic studies, (2) designing and accelerating shotgun proteomics experiments, (3) confirming or questioning gene models, and (4) adjusting gene models such that they are in line with observed *Drosophila *peptides. While the database consists of proteomic data it is not required that the user is a proteomics expert.

## Background

In 1995 the first complete genome sequence of a species was published [1]. Since then, the genomes of many other species have been sequenced and an increasing number of technologies have been developed that measure at a large or genome-wide scale specific properties of the genome and the products derived from it. These data increasingly complement the molecular biological approaches that are focused on specific molecules [2]. These so-called *omics *technologies are driven by measurements of thousands of molecules at a time. Due to the production of large amounts of complex data, such studies challenge data organization and management and call for bioinformatic contributions to biology.

In the case of mRNA measurements, microarray technologies generated the first data sets more than a decade ago, and today the corresponding computational needs have largely been met. In the case of proteins, however, quantitative high-throughput measurements remain a challenge. The wide range of protein concentrations in cells and tissues and other complications cause significant challenges for the comprehensive quantitative measurement of a proteome, one of which is the undersampling of the peptides generated from complex protein samples. In 2006, the technology of Selected Reaction Monitoring (SRM, also referred to as MRM or mSRM) has been proposed as a method for global proteome quantification that has the potential to alleviate some of the limitations of the current proteomic strategies [3,4]. The key solution provided by SRM to the complexity challenge of the proteome is the quantitative, directed survey of known and previously observed protein-specific peptides (so called proteotypic peptides, PTPs). The required cataloging of PTPs and their mass spectra poses an experimental as well as a bioinformatic challenge and is a pre-condition of any SRM assay development – much like the sequencing and assembling of whole genomes was for microarray studies. Once the PTP catalogs are established and accessible, they enable biologists to confidently quantify the expression of many proteins in parallel with high sensitivity and throughput [5].

One underlying requirement for proteomics experiments of any kind, but particularly for directed strategies, are correct primary sequences of an organism's proteins and their splice forms. Owing to *Drosophila melanogaster*'s status as a well established genetic and molecular biological model organism, its genome was sequenced early on [6,7] and improved genomic builds continue to be released [8-17]. Alongside with the genomic sequencing, there have been several large-scale efforts to produce high quality cDNA libraries [18,19]. As a result, the annotation of the fly genome is well developed compared to other organisms. However, there are still gene models that are based on computational predictions only or for which the cDNA/EST data are incomplete. Therefore, it is advantageous to use protein data as an additional source of information for the annotation of protein-coding genes [20-24].

To be useful for a task as important as genome annotation, raw proteomic data have to be processed to the point of tightly quality controlled peptide identifications and organized in a suitable meta-level structure. To meet this need, we present the *Drosophila melanogaster *PeptideAtlas. It is a resource of *Drosophila *peptides we observed experimentally. The atlas facilitates access to peptide sequences and their corresponding mass spectra to any researcher interested in *Drosophila melanogaster *not requiring that the user is a proteomics expert. More precisely, using a local version of the publicly available database systems SBEAMS and PeptideAtlas as well as the HTML-based user interface [25-27] the results of a large number of diverse shotgun proteomics experiments [28] were warehoused. All tandem mass spectrometry (MS/MS) data were uniformly processed into a master list of observed *Drosophila *peptides mapped to the genome and a representative consensus mass spectrum has been computed for each peptide's charge state and modification. For visualization of the peptides in genomic context, the data are integrated with the FlyBase genome browser.

By presenting this – observed – part of the otherwise mostly predicted proteome along with important features, such as direct FlyBase connections, consensus spectra, and peptide annotation with observed modifications, the PeptideAtlas interfaces well with the tools commonly used by the *Drosophila *community. In this way, it contributes to the design of targeted proteomics experiments as well as to improved genome annotation. In this manuscript, we show how information stored in the PeptideAtlas can be accessed and used to confirm and interrogate gene models as well as for improving future proteomics experiments and their interpretation.

## Construction and Content

### PeptideAtlas construction workflow

**Figure 1:**
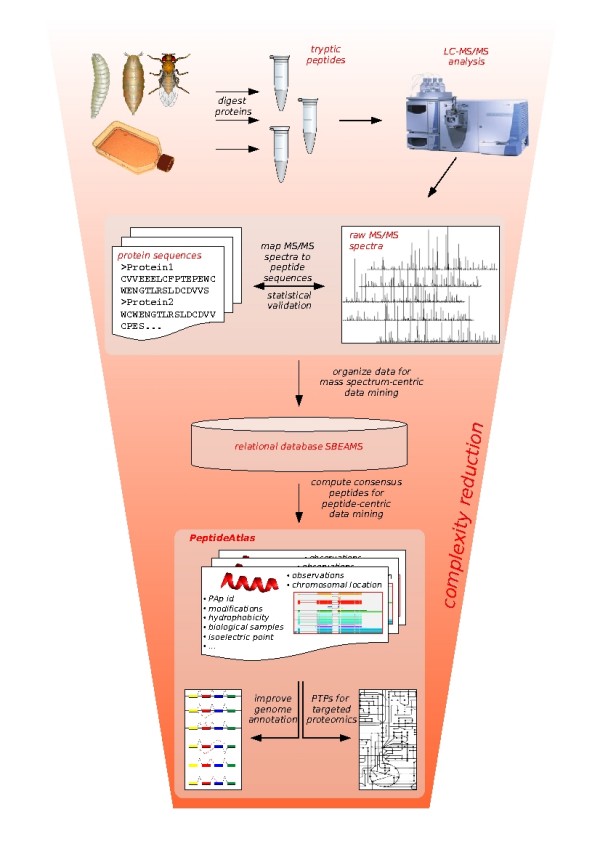
**The placement of PeptideAtlas**. The placement of PeptideAtlas bridging global and targeted proteomics experiments is depicted. Protein extracts were prepared from diverse *in vivo *and *in vitro *samples. The proteins were digested and, after additional fractionation steps, analyzed via LC-MS/MS. The spectra were mapped to peptides that likely gave rise to them using sequence database searching. Subsequently, the mappings were statistically validated. Spectral centric data mining was facilitated by a relational database (SBEAMS). Then, re-organization of the data in a peptide-centric manner was the focus during PeptideAtlas construction: Data accumulated over many experiments, generated over the course of several months or years, was complexity-reduced and condensed concentrating on the actual peptide entities. This now allows for retrieval of high quality proteotypic peptides as well as for gene model validation based on expressed peptide sequences.

Aiming for a structured compendium of the *Drosophila melanogaster *peptides observable by mass spectrometry (MS), fly proteins were analyzed experimentally and computationally as depicted in Figure 1. For each identified peptide, all observation instances with a probability for a correct assignment *p *≥ 09 were coalesced into a PeptideAtlas peptide (PAp) entity. The PAps are the core entities of the PeptideAtlas database. In the following, the steps of the PeptideAtlas generation workflow are described in detail.

### Data generation and processing

#### Protein extract preparation and mass spectrometry

Protein extracts from *Drosophila *cells, tissues, and organelles were analyzed in a typical shotgun proteomics workflow via liquid chromatography and subsequent ion trap tandem mass spectrometry (LC-MS/MS). For most biological experiments, the sample preparation was performed as described thoroughly in a previous publication [28]. The additional data analyzed in this study consist of 9 experiments in which Kc167 cells were studied. They were grown to high density in 150 cm^2 ^flasks in Kc *Drosophila *cell culture medium (Invitrogen) supplemented with 10% FCS. The cells were harvested and then transferred to 50 ml Falcon tubes and centrifuged (1000 g, 7 min, 4°C). The pelleted cells were washed 3 times with 50 ml of chilled PBS by using the initial centrifugation conditions. The washed cell pellet was resuspended in 5 volumes of fresh ice cold hypotonic cell lysis buffer [10 mM Hepes (pH 7.90), 1.5 mM MgCl_2_, 10 mM KCl] supplemented just before use with 0.5 mM DTT and complete protease inhibitor cocktail (Roche) and incubated in this solution for 10 min on ice. The swollen cells were bounced 20 times or until all cells were visibly lysed. Mass spectrometry analysis was performed on an LTQ mass spectrometer as explained in [28]. In total, from 55 experiments consisting of 1'788 LC-MS/MS runs, 8'529'853 fragment ion spectra (MS2 spectra) were acquired.

#### Assignment of peptide sequences to mass spectra

Once the peptide mass spectra were acquired, spectra with a minimum peak count of 5 and with a precursor ion m/z value within the window of [800, 4200] Thomson (Th) were selected [29]. Those 7'307'708 MS2 spectra were sequence database searched against the Ensembl protein database v27.3c to identify the corresponding peptide sequences. In addition, the spectra from a subset of 4 experiments were searched against the Ensembl genome database v31.3e translated in all 6 reading frames. FlyBase is the canonical reference database for the *Drosophilidae*. Ensembl does not perform its own gene predictions for *Drosophila melanogaster *but instead serves the FlyBase annotations connected to the large variety of Ensembl services, such as interfaces for programmers. Please note that FlyBase annotations were retrieved through Ensembl due to technical reasons. PeptideAtlas makes use of the Perl API offered by Ensembl. Table 1 gives an overview of the sequence database releases used in this study.

**Table 1:** Databases used in this study.

	Ensembl	FlyBase	
	Eu- and Heterochromatin	Euchromatin	Heterochromatin
Search against protein database	v27.3c	r3.2	-
Search against 6-frame translation of DNA	v31e	r3.2.1	-
Reference database for coordinate resolution	v42	r4.3	r3.2b2

Listed in the different columns are the Ensembl database releases used for searching and the FlyBase/BDGP releases they correspond to.

The searches were performed employing TurboSEQUEST v.27 [30] allowing fully tryptic peptides (cleavage C-terminal of lysine and arginine, unless followed by proline) with up to 2 missed cleavages. Precursor ion m/z tolerance was set to 3 Th and cysteine alkylation as a static modification and variable methionine oxidation were included. To control the identification error rate, probabilities for correct assignments of peptides to mass spectra were computed applying an empirical statistical model of assignment score distributions using the algorithm PeptideProphet [31] and protein identifications were inferred using the ProteinProphet program [32]. As a control for *Wolbachia *contamination, protein extracts were also searched against the protein database of *Wolbachia pipiensis *TIGR v18. No *Wolbachia *proteins were found.

#### Processing of identified MS2 spectra

In the above-described manner, 576'468 peptides were identified with an estimated maximum error rate of 3.17%. They correspond to 76'724 distinct peptide sequences. For each of the peptides identified by this process we computed standard value sets to generate PeptideAtlas peptides. Each PAp is characterized by a series of properties: its sequence, its isoelectric point, its relative hydrophobicity [33], its number of observations, as well as the modification- and charge states and sample(s) in which it was observed.

The peptide sequences were aligned with the *Drosophila melanogaster *Ensembl protein database v42 (cf. Table 1) using the blastp algorithm available through the blastall program v2.2.14 with a SEG low complexity filter and a PAM30 scoring matrix. Word size was 2, gap penalty 9, and gap extension cost 1 [34]. Subsequently the coordinates of peptide mappings to the genome were retrieved using the Ensembl Perl module Bio::EnsEMBL.

### Relational database, its user interface, and data loading

An instance of the database system SBEAMS [35] was set up locally. It consists of a database backend running several databases on one MSSQL Server 2000 and a HTML-based front end. The latter one is generated through Perl Common Gateway Interface scripts operated on an Apache 2 server running on a Debian Linux server. The open source implementation of the Tabular Data Stream protocol FreeTDS serves as an OS-bridging interface between backend and frontend.

The results of the above described data generation and processing steps were then loaded to the database: All PAp entities and the genomic locations they mapped to were imported into the database module PeptideAtlas. For details of its relational schema, please refer to [36].

## Utility and Discussion

### Coverage of the proteome

In total 9'263 protein isoforms and 8'799 gene models (65%) of sequence database v27.3c are represented in this PeptideAtlas database having a sequence coverage ranging from less than 1% up to 100%, with an average of 25%. More specifically, 52 protein isoforms have more than 90% of their primary sequence covered, 2 of which are fully covered (CG4800-PA, encoded by *Tctp*, and CG4918-PA/-PB, encoded by *RpLP2*). 19 proteins (52 isoforms) are each covered by more than 100 peptides. The most extreme case is the protein CG1915-PC (*sls*) for which we observed 262 peptides (31.4% of its partly repetitive 18'074 amino acids sequence translated from 37 exons). The established *Drosophila *PeptideAtlas is a source of information about 76'724 peptides and provides an empirical footprint (i.e. observed quantity and characteristics) for each peptide entry. The largest PeptideAtlas, the *Homo sapiens *build [37], covers 22'983 gene models (ca. 52% of all Ensembl v43 genes). The fly build introduced here is slightly smaller than the human one. However, it actually has a gene model coverage that is 13% higher than the one of its human counter part.

Expression constraints such as developmental stage, environmental conditions, or tissue specificity are not know for the majority of proteins. Thus, during the protein extract preparation a directed large-scale approach was used which combined different fractionation techniques with large biological sample diversity. We iteratively targeted protein groups with different physicochemical and functional parameters and expression ranges. The achieved coverage of 65% of the fly gene models therefore is a result of intensive efforts to reach high proteome coverage. A strategy to significantly increase the coverage has been proposed and shown to work [28,38]. However, proteins corresponding to 35% of the gene models have not been observed. Several other research groups describe similar experiences when targeting whole proteomes of other organism and difficulties associated with full proteome coverage have been acknowledged [28,39,40]. Next to wrong gene models, reasons for missing 35% of the fly's genes can be the broad dynamic range of protein expression, instable proteins, and the lack of either expression or of analysis of a specific biological condition. Generally, a protein might simply be expressed under the detection limit. Some limitations of the shotgun proteomics strategy may also contribute to this lack of observed peptides. These include undersampling due to highly complex protein samples, non-suitability of peptides for the employed ionization method, imperfect scoring schemes of sequence database search engines and statistical postprocessing, posttranslational or other chemical modifications of peptides not anticipated during the MS2 spectra identification, and unanticipated peptide fragmentation.

As more fly protein extracts will be analyzed, we will update the atlas regularly. This PeptideAtlas is intended as a resource from and for fly community members. Any researcher who is willing to share their fly protein mass spectrometry data with the community, and thereby enhance fly research in the fields of genomics and proteomics, can use the Atlas as a tool to do so. We advocate the contribution of other laboratories to the next PeptideAtlas release. The use of standards for data quality and reporting ensures a user can actively use data from other laboratories without proteomics expert knowledge. The identification pipeline presented here, with its core being the combination of TurboSEQUEST and PeptideProphet, is a well-established and widely accepted data analysis procedure that ensures high data quality and consistency.

### Utility of PeptideAtlas and associated tools

PeptideAtlas has several features that support biological disciplines important for studies on a large (systems) level, such as mass spectrometry-based proteomics and genome annotation.

#### The fly PeptideAtlas supports proteomics in *D. melanogaster*

##### PeptideAtlas as a resource for shotgun proteomics employing spectral library searching

Since the beginning of shotgun proteomics, peptide identification has been based almost entirely on sequence database searching, in which the fragment ion spectra generated by a tandem mass spectrometer are compared to lists of spectra predicted from the sequences in a protein database. Recently it has been shown, that significantly improved sensitivity, specificity, and speed of peptide identification can be achieved if pre-acquired spectra are available and are processed into consensus spectra (weighted combinations of multiple spectra of the same peptide [41]) to which the collected fragment ion spectra are matched [42]. The information present in a consensus spectrum is both more sensitive than theoretically computed spectra as it builds on experimental knowledge, and more robust than single observation spectra since the consensus spectrum building process reduces or eliminates noise. During consensus spectrum calculation the typical relative fragment ion signal intensity is determined empirically and hence can be taken into account during the spectra comparison. We have generated consensus fragment spectra for the peptides contained in the fly PeptideAtlas. They were compiled from conventional sequence database search results in which the fragment ion spectra in the fly data set were searched sequentially with the search engines Sequest, OMSSA, X!Tandem, and Protein-Prospector/Batch-Tag [43-46]. For the computation of consensus spectra, the spectra were clustered using the dot product as similarity measure [41,47]. Peaks present in the majority of replicate spectra of a cluster were included in the consensus spectrum with their abundance calculated based on weighted averages of peak intensities (see additional file 1: Text explaining the consensus library construction). The resulting library consists of 65'503 entries.

To benchmark searching our library, we assessed its performance in a typical use case. A new data set of fragment ion spectra was generated and searched in two ways: 190'948 MS2 spectra of the cytoplasmic fraction of *Drosophila *Kc cells which had not been part of the library construction were searched in two ways: (1) in a classical sequence search (Tandem k-score, Ensembl v27.3c) and (2) searching our consensus library employing the software tool SpectraST [42]. In both cases, the results were statistically validated using PeptideProphet and it was filtered at an estimated error rate of ≤ 0.005. In the classical case, we were able to identify 2'946 peptides (914 are not present in the spectral library) based on 4'050 spectra. When performing spectral library searches, we identified 4'603 peptides based on 9'332 spectra (data not shown). This demonstrates of the usefulness of our consensus library. Searching this library is more sensitive and specific than traditional sequence database searching. In addition, the spectral library searching has been shown to be considerably faster than traditional sequence database searching [42]. Thus, it is a very useful and effective way to make use of the information served by the *Drosophila *PeptideAtlas. *Drosophila *researchers planning to perform fly proteomics experiments can greatly benefit from the pre-acquired data compiled in PeptideAtlas, since they can now identify peptides that are part of this library very quickly by spectral library searching. During the subsequent analysis, they can then concentrate on the yet unidentified spectra. The consensus spectra are available via the database's user interface. The figure in additional file 2 shows a typical user session employing the graphical user interface of PeptideAtlas (see additional file 2: Explanatory screenshots of a PeptideAtlas session using the HTML interface).

When performing spectral library searches, for a peptide to be identified its combination of charge state and modification has to be part of the library. The spectral matching approach can therefore only be as complete as is the underlying employed collection of consensus spectra. With an increasing number of peptide observations, there is an increased probability that an observation of a peptide with a given modification and charge state has been made before. The consensus spectra collection we present is the most comprehensive one offered for *Drosophila melanogaster *known to the authors. As new peptides will be added to the PeptideAtlas in the future, we will continue to upgrade and extend our collection.

##### PeptideAtlas as a resource for targeted proteomics using proteotypic peptides

To date, most large-scale qualitative or quantitative proteomics experiments are carried out by shotgun mass spectrometric measurements of tryptic digests of the respective samples. Such data sets are characterized by a large redundancy and other limitations discussed above. With the emerging proteomics technology based on SRM [3], it is now possible to reach a high throughput for targeted experiments as well [38]. Specifically, it is possible to probe for specific proteins and measure protein expression quantitatively on a large scale at significantly reduced redundancy [5,48]. However, in order to perform efficient SRM experiments, three requirements must be fulfilled: One needs to know (i) the PTPs of the proteins of interest, (ii) in which charge state and with which modifications a PTP is observed *in vivo*, and (iii) the spectra actually observed in the triple quadrupole mass spectrometer used for such measurements. This helps in choosing suitable precursor ion to fragment ion transitions [49]. The first question, i.e. which peptides are specific for a given protein and, in addition, are well detectable within a multidimensional separation mass spectrometry experiment can be answered most reliably by observing protein-specific peptides in the MS in multiple repeated experiments. The PeptideAtlas database provides this by offering compiled information about the frequency and quality of observations of PTPs. The second question, which modifications a peptide carries *in vivo *and the MS ionization charge state of the peptide, can also be best answered through many repetitive experiments and observations. PeptideAtlas provides these answers, too, by making accessible the observed PTPs and their spectra. We meet the third need, i.e. to know a typical mass spectrum of a PTP, by providing not only the individually observed spectra, but also the computed consensus spectra. The figure in additional file 2 illustrates step by step how one can query the Atlas using its HTML interface. For any given peptide, the user can retrieve the corresponding consensus spectrum and download it for local use (see additional file 2: Explanatory screenshots of a PeptideAtlas session using the HTML interface). This can then be used as a high quality starting point for the validation of SRM transitions.

#### PeptideAtlas supports validation of models for protein-coding genes

##### Visualization of the peptides in the FlyBase genome browser

**Figure 2:**
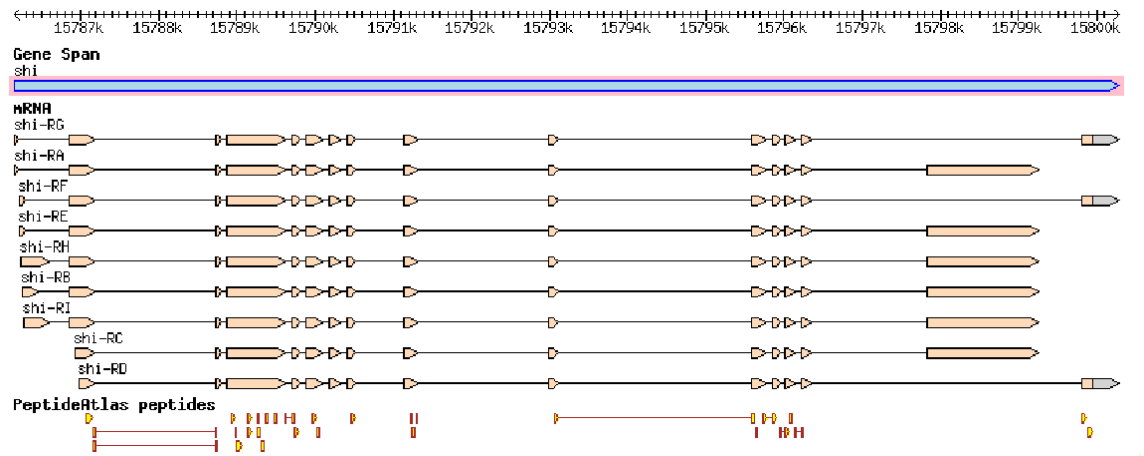
**GBrowse on the FlyBase website**. The figure shows a screenshot of the genome browser GBrowse on the FlyBase website. The gene model of *shibire *(*shi*) on the forward strand of the X chromosome is currently known to have 9 different mRNAs encoding 2 different proteins. 29 peptides are displayed that map to only one location in the genome (*shi*) and have been observed at least twice. 23 of these peptides lie within exons. In addition, 6 peptides lie across 2 exons and cover 5 splice sites. The peptides are hyperlinked to the PeptideAtlas website where more information about each peptide is available.

For straightforward exploitation of gene model confirming peptides, they were integrated into the FlyBase database. In this canonical data source for *Drosophila*, the peptides can be browsed using the popular genome browser GBrowse. Figure 2 shows the relation of PeptideAtlas peptides and the FlyBase annotations as users can now browse it. All peptides in the genome browser are hyperlinked to the PeptideAtlas website where more information about each peptide and its observations is available. Hence, within the context of the other types of information served by FlyBase, such as cDNA or EST coverage, transposon mappings, currently annotated introns and exons, etc., it is now, for the first time, apparent to the user which parts of a predicted protein have actually been observed in wet lab studies.

##### Confirmation of gene models

Besides gene predictors, cDNA, and EST data, the additional complementary consultation of protein data is invaluable for a correct genome annotation. Protein data has two main strengths over nucleotide sequence data: Ultimately, only protein evidence can answer the questions of which splice donor and acceptor sites are spliced together in the final mRNA splice form and which final transcript is actually being translated. In addition, definite knowledge about the frame in which a transcript is being read can only be derived from the translation products, the proteins. For this reason, we conducted a systematic analysis of the PeptideAtlas data with respect to the annotation of gene models: Here, all PAps have been mapped to a protein database more recent than the one used for original identification of the peptides, namely the Ensembl release v42 (cf. Table 1). This resulted in coverage of 13'802 isoforms (9'044 gene models) and confirmation of 6'221 splicing events. SwissProt, which is widely accepted as the protein database of highest quality currently lists 1'552, fly protein sequences as supported by protein evidence. The data reported here therefore raise this number significantly by contributing mass spectrometry-based peptide evidence for an additional 7'746 proteins. This provides important, validated information for *Drosophila *biologists. For protein sequence annotation, the peptide data complements the nucleotide sequence alignments that have been used exclusively in the past. To prove the occurrence of a predicted splicing event and that the resulting transcript is translated, a peptide covering the splice site is both necessary and sufficient evidence. However, due to the short lengths of peptides there is an appreciable frequency of information loss about the origin of an observed peptide; thus, in shotgun proteomics it is usually not possible to prove the existence of a specific isoform along its entire length.

##### 'Lost' peptides

**Figure 3:**
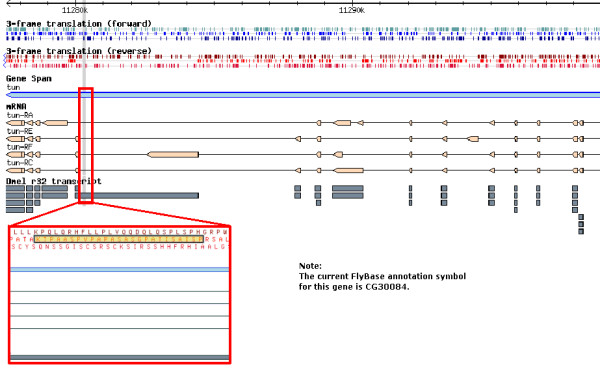
**A 'lost' peptide in CG30084**. Shown here is part of the gene CG30084 (it was called *tun *in release 4) of the FlyBase annotation. Within the FlyBase release 3.2 it was annotated to encode the 4 protein isoforms CG30084-PA, CG30084-PB, CG30084-PC, and CG30084-PD (shown in dark grey). The PeptideAtlas peptide PAp00061581 (PSIASITAPGSASAPAPVPSAAPTK) was part of the splice variant CG30084-PB (red frame, PAp00061581 highlighted in yellow). The annotations of the subsequent release 4.3 are shown in beige color. As one can see, none of the 4 isoforms in this newer release (CG30084-PA,-PC,-PE, and -PF) can account for the observed peptide.

Interestingly, 81 peptides identified based on version v27.3c of the Ensembl protein database were 'lost' with progressive updates on the sequence database, i.e. they could no longer be mapped to version v42 (cf. Table 1). Depicted in Figure 3 is an example case of what we call 'lost peptides': The PeptideAtlas peptide PAp00061581 with the amino acid sequence PSIASITAPGSASAPAPVPSAAPTK has been observed 9 times (in the charge states 2+ and 3+). In the initial database search it was identified as part of CG30084-PB, a protein involved in cytoskeleton organization. However, the peptide is 'lost' in the later release v42, i.e. it is not part of any isoform of CG30084 or any other annotated protein in the more recent release. The locus this peptide was originally assigned to is located on the reverse strand of chromosome arm 2R and was, according to the database release v27.3c, annotated to have 4 different isoforms (CG30084-PA, -PB, -PC, and -PD, FlyBase annotation release 3.2). The 'lost' PAp was encoded in the splice variant CG30084-PB. However, in the subsequent release v42, partially different splice forms were annotated (CG30084-PA,-PC,-PE, and -PF, FlyBase annotation release 4.3). With those annotations, the peptide could no longer be explained. This shows that this particular gene annotation actually deteriorated with this newer release. However, based on our peptide data, we confirm the annotation in the even more recent FlyBase annotation release 5.2 to now be correct. There, the 4^th ^exon of isoform CG30084-PF was changed and the annotation is now inline with the peptide data.

Due to the continuous, invaluable manual work of the FlyBase gene model curators gene models continue to change. However, there is no assurance that the latest annotations are always correct. Here, we have shown how the information in the PeptideAtlas can help this process by providing an additional source of support for potential gene models. In cases for which other supporting data are weak or absent, such as the CG30084 transcript described above, peptide data are especially important.

Based on a comprehensive data set which includes sequences, assemblies, and alignments of the genome sequences of 11 additional *Drosophila *species [50,51] Lin and colleagues developed new predictions for the fruit fly's exons [17]. Interestingly, based on conservation, they predict exons suggested also through an additional 72 of the 80 'lost' peptides. FlyBase recently corrected the underlying gene model annotations, such that the latest gene models are now in line with many exons predicted by Lin et al. and those 72 'lost' peptides detected in this study.

There is no other genus known to the authors where there is or will be in the near future such a rich comparative data set as basis for exon predictions. Thus, we argue that for other species, it is advisable to generate a PeptideAtlas for substantially improving genome annotation, as the gain from such an enterprise is very likely to be even higher in other species compared to *Drosophila*.

Notably, 8 peptides remain 'lost' (cf. Table 2). Those peptides could be false positive identifications; this number does lie well within our error rate of 3.17%. However, since, like the 72 cases described above, these 8 peptides were previously in gene model context, they may be additional cases of errors introduced in subsequent annotation releases. They will be the subject of future reassessment by FlyBase annotators.

**Table 2:** Table listing the 8 "lost" peptides which are not in line with the latest annotation.

PA accession	Peptide sequence	No. of observations
PAp00063021	RIINFGSNHTANTATKALGAGSEAGAGAGVGMATATATATVGR	1
PAp00058607	MELHKQYTTVGASMLTPPDAKAIIAGPTDLYVK	1
PAp00060480	NNAPGLINAGIVELDSHNLILAR	1
PAp00053627	ISMHSAAICPPGAR	1
PAp00068376	TTTTDGAIRR	3
PAp00047403	ERERTTIVR	4
PAp00051256	HEVAVGAEQGGADNLR	1
PAp00071978	YPLYYTVHSAPEQHHIHVYHLPVCK	1

##### Six-frame peptides

**Figure 4:**
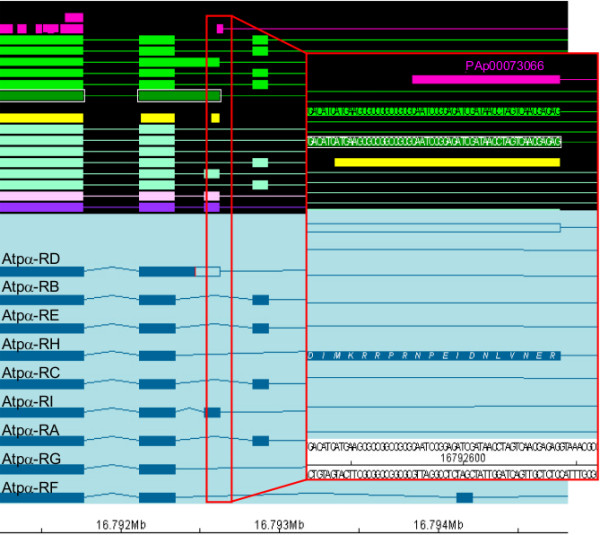
**A peptide highlights a missing splice form**. Part of the gene model of the Na pump alpha subunit (Atpα, CG5670) is depicted. In front of the black background, different types of sequence data are displayed: several predictions (light pink, purple, and different shades of turquoise), conserved coding regions (bright yellow), cDNAs alignments (greens), and peptides from the PeptideAtlas (bright pink). In front of the light blue background, alternative splice forms annotated in release 5.12 are shown in dark blue. The PeptideAtlas peptide PAp00073066 was identified in a 6-frame search and maps within the Atpα gene region. Note that while prediction algorithms postulate an alternative exon in this region, there are no supporting cDNAs (nor ESTs; not shown). The splice variant Atpα-PI, added in FlyBase annotation release 5.11, now accounts for the identified peptide sequence, NPEIDNLVNER. The codon for the last residue of the peptide spans the adjacent intron, thus supporting the annotated splice sites.

Aiming to find peptides not anticipated by the current genome annotation, we searched a subset of the PeptideAtlas data against a 6-frame translation of the genome. A set of 889 distinct peptides originally found in this genomic search were not in agreement with any gene model annotated in the reference database. Those sequences can potentially represent currently un-annotated stretches of expressed sequence or novel splice variants. To understand the origin of those matches, we further investigated their genomic context. In a first step, we looked for peptides that could be explained by a newer release of FlyBase. We found that 68 peptides were explained by the newer annotation r5.2 or encoded by transposons. In a second step, to avoid ambiguous genomic placements, peptides were excluded which matched to more than one genomic location. As a third step, those peptides were filtered out which had been identified based solely on mass spectra in which no fragment ions with a larger m/z value than their precursor mass were observed (therefore likely representing singly charged peptides which often deliver poor spectra). Lastly, all spectra with a quality value < 1 as computed by the algorithm Qualscore [52] were removed from the final set. The remaining 250 peptides were subjected to detailed manual analysis in collaboration with the FlyBase curators. It was found that 46 peptides point to conservation-based exon predictions. By using the peptides, gene models that are likely to benefit most from those predictions can be identified easily by pointing the curators to predictions that can be confirmed with peptide data. An example case is shown in Figure 4. The peptide NPEIDNLVNER supports the addition of a novel isoform of the Na pump α subunit (Atpα, CG5670). In this case, several different prediction algorithms postulate a unique exon, but there are no cDNA or EST data to support such an alternative transcript. The peptide data confirm that the exon in question exists. Overall, new potential splice variants have been generated and will be included in a future FlyBase release. The remaining peptides partly contradict other evidence. They could potentially represent small genes, novel exons, cases of alternative reading frames, or false positives. They are subject to ongoing investigations. This shows that, even in *Drosophila melanogaster*, a species that is likely to have one of the best annotated genomes amongst higher eukaryotes, the use of PeptideAtlas information leads to an improvement of the genome annotation.

## Conclusion

In this work, we established the *Drosophila melanogaster *PeptideAtlas, a resource of observed mass spectra matching to 76'724 fly peptides and we show it use and potentials.

The data in the atlas confirms gene models for 9'263 protein isoforms on the amino acid level, questions some existing gene models, and provides evidence for the introduction of new transcripts. Besides the PeptideAtlas' application to confirmation of gene model architectures and its use to identify necessary changes in the genome annotation, it is a valuable resource for proteomics. PeptideAtlas provides protein-specific peptides and the consensus mass spectra that represent them. By making the fly proteome we detected by mass spectrometry available to the research community in such a peptide-centric manner, qualitative and quantitative proteomics experiments in *Drosophila *are greatly facilitated. Specifically, the availability of consensus fragment ion spectra supports the identification of *Drosophila *mass spectra by spectral library searching with greater speed, sensitivity, and specificity than was possible in the past.

Overall, the PeptideAtlas serves as a data-mining tool and enables the user to mine the valuable information present in mass spectrometry data from experiments contained in the largest (and growing) *Drosophila *proteogenomics data set known to the authors. It provides not only the confirmation that a peptide is observable, but also the specifics of where, when, and in which form it is observed. Thus, the *Drosophila *PeptideAtlas facilitates improved genome-scale studies central to modern biology.

## Availability and Requirements

The *Drosophila melanogaster *is available free of charge at the web address .

## List of abbreviations

BDGP: Berkeley *Drosophila *genome project; DHGP: *Drosophila *heterochromatin genome project; ICAT: isotope-coded affinity tag; LC: liquid chromatography; LTQ: linear trap quadrupole; MS: mass spectrometry; MS/MS tandem mass spectrometry; MS2 spectrum: fragment ion mass spectrum; PAp: PeptideAtlas peptide; PTP: proteotypic peptide; SRM: selected reaction monitoring (old terms: MRM: multiple reaction monitoring; mSRM: multiple selected reaction monitoring); TIGR: The Institute of Genome Research.

## Authors' contributions

S.L. conducted most of the work and wrote the manuscript. E.B. provided experimental data, in addition to ideas, and helpful discussions. N.K. and E.D. helped in installing PeptideAtlas. S.S. constructed the consensus library. E.H. and R.A. held senior authorship responsibilities.

## Supplementary Material

Additional file 1 **Text explaining the consensus library construction** Click here for file

Additional file 2 **Explanatory screenshots of a PeptideAtlas session using the HTML interface** Click here for file
