# Supplementary material for: The Drosophila melanogaster PeptideAtlas facilitates the use of peptide data for improved fly proteomics and genome annotation
Source: BMC Bioinformatics. 2009 Feb 11;10:59. doi: 10.1186/1471-2105-10-59 (PMC2648944; doi:10.1186/1471-2105-10-59)
Supplement: Additional file 1 — Text explaining the consensus library construction [file 1471-2105-10-59-S1.pdf]

“The *Drosophila melanogaster* PeptideAtlas facilitates the use of peptide data for improved fly proteomics and genome annotation.”

Additional File 1

Spectral library construction

For the construction of the spectral library, the assignments of spectra to peptides were computed independently of those ones for the actual PeptideAtlas construction. They were computed by the sequence search engines Sequest, OMSSA, X!Tandem, and Protein-Prospector/Batch-Tag. Reported scores for these search engines were individually normalized using results of searches against a combined forward and reversed sequence collection. The greatest derived probability (or expectation value) among the search engines was used in further processing. Parent and fragment ion tolerances of 2 and 0.8 Th, respectively, were generally used. Charge states of at least +1 to +3 were examined for all spectra. Variable methionine oxidation, N-terminal acetylation, glutamine deamination, and fixed cysteine alkylation were used as modifications. Peptides with asparagine deamidation were identified (to remove them as potential interference).

Multiple spectra assigned to a single peptide ion were then combined to form a consensus spectrum. This involved the following series of steps for each identified peptide ion: (a) Identify spectra with the highest sequence search scores (maximum of 100 spectra). (b) Align m/z values in the different spectra (0.1 m/z bins are used). (c) Compute spectrum similarity dot product [32] for each spectrum pair. (d) Identify cluster of most similar, highest scoring spectra, reject the rest. (e) Include peaks in the consensus spectrum that were in the majority of replicate spectra that had sufficiently high signal/noise (S/N) values for those peaks to have been observed. (f) Generate an abundance from weighted averages of peaks using the square root of computed S/N for that

spectrum as the weighting factor (S/N is taken as the ratio of maximum to median abundance in a spectrum). Abundances were given as integers after base peak normalization to 10000. (g) Compute measures of overall spectrum and individual peak variation. Product ions peaks were labeled using conventional y-, b-, a-type notation, along with immonium ions, internal ions (from singly charged precursor ions only), and common neutral loss ions, including losses from the parent ion. Up to 2 assignments were given for each m/z, using simple rules for ordering which include proximity to the expected m/z and ion type with a 0.8 m/z tolerance. With consensus spectra, additional information is given that describes the variability of the peaks among the underlying replicate spectra. Using original sequence search scores, parent m/z accuracy, and fraction of unassigned abundance, a best replicate spectrum was identified. In cases where no good consensus spectrum could be generated due to loss of significant peaks in the averaging process, this spectrum was the only representation for a peptide ion. In cases where only highly impure replicate spectra were available, they were omitted; otherwise the best replicate spectrum was selected for each consensus spectrum.

Following creation of consensus spectra and selection of best replicates, a series of quality control steps was applied to refine the probability of correct identification and to limit spectra dominated by impurity peaks and from homologous peptides. Beginning with the highest probability of correct identification derived from search engine results, the probability was refined using the following factors: (a) Accordance of y/b ions with theoretical spectrum. The theoretical spectrum was derived from estimated relative cleavage rates for each pair of adjacent amino acids. These rates were derived from a collection of reliably identified peptide spectra and were divided into two classes (with/without mobile protons). (b) Fraction of unassigned abundance of the largest 20 peaks. This included peaks that could not be explained as y-, b-, or a- ions, internal ions, or ions derived from common neutral losses. (c) Continuity of y- or b-ion series. Longer series of contiguous y- and b-ions increased probabilities of correct identification. (d) Number of peptide

ions with the same sequence, including different charge states and modifications. Corrections were derived by comparing results for FP identifications (from reverse library searching) to TP results at the same sequence search engine score. Spectra were included in the library when the final quality score was above a pre-set threshold. These threshold values were set so that the likelihood of being correct was >95% and the quality measures indicate that it has a suitably high S/N and low impurity content for it to reliably serve as a referee. A tryptic peptide with no missed cleavages, for example, had the lowest threshold for acceptance. At the other extreme, all semi-tryptic peptides with unexpected missed cleavages were rejected. To avoid 'homology' errors (some correct product ions, but wrong precursor), extra penalties were also applied to non-tryptic peptides that had higher levels of unexplained peaks. These settings were based primarily on results from digests of known proteins, where it was found that false matches with high scores were generally such 'homologous' peptides. As a final step, all similar spectra with similar precursor m/z values were inter-compared, and for sets of similar spectra (dot product > 0.7), one spectrum was selected. In this case, tryptic peptides were preferred over semi-tryptic, significantly higher scoring spectra favored over lower and spectra with more source spectra favored over those with fewer.
