# Supplementary material for: The Drosophila melanogaster PeptideAtlas facilitates the use of peptide data for improved fly proteomics and genome annotation
Source: BMC Bioinformatics. 2009 Feb 11;10:59. doi: 10.1186/1471-2105-10-59 (PMC2648944; doi:10.1186/1471-2105-10-59)
Supplement: Additional file 2 — Explanatory screenshots of a PeptideAtlas session using the HTML interface [file 1471-2105-10-59-S2.pdf]

“The *Drosophila melanogaster* PeptideAtlas facilitates the use of peptide data for improved fly proteomics and genome annotation.”

Additional File 2

Explanatory screenshots of a PeptideAtlas session using the HTML interface

This figure shows an exemplary PeptideAtlas user session at www.drosophila-peptideatlas.org and is to be read clockwise. The user wants to know a proteotypic peptide for the Heterogeneous Nuclear Ribonucloprotein Hrb87F (CG12749), more specifically its splice form CG12749-PA. **Upper left:** Result of a Protein Query for CG12749-PA: a condensed representation of the protein and its aligned PAps. **Upper right:** In the Peptide Query, one can narrow down the search by allowing only PAps specific for this gene and, even further, the splice variant CG12749-PA. In addition, the constraint is applied, that a peptide has to be observed at least twice. This way, PeptideAtlas pinpoints to 2 of the 44 peptides one of which (PAp00011594) has been observed 62 times. Both PAps would typically be chosen as PTPs for the design of an SRM assay. **Lower right:** Clicking on a PAp accession provides detailed information about this peptide. The 64 observations divide into 16 in the charge state 2+ and 46 in 3+. **Lower left:** In particular, one can view and download consensus spectra for either of those charge states.

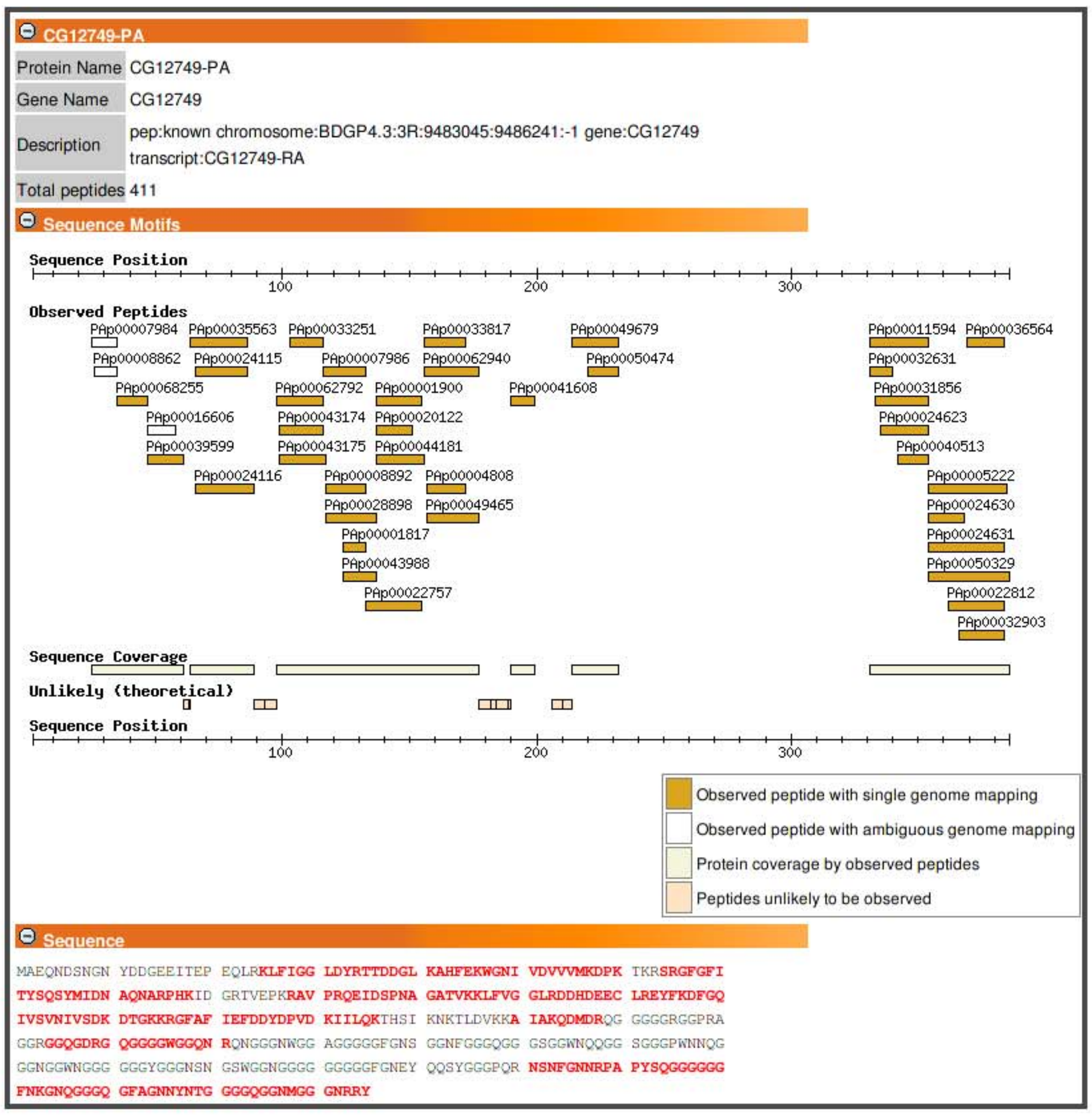
CG12749-PA
Protein Name CG12749-PA
Gene Name CG12749
Description pep:known chromosome:BDGP4.3:3R:9483045:9486241:-1 gene:CG12749 transcript:CG12749-RA
Total peptides 411
Sequence Motifs
Sequence Position
Observed Peptides
Sequence Coverage
Unlikely (theoretical)
Observed peptide with single genome mapping
Observed peptide with ambiguous genome mapping
Protein coverage by observed peptides
Peptides unlikely to be observed
Sequence
MAEQNDSNGN YDDGEEITEP EQLRKLFIGG LDYRTTDDGL KAHFEKWGNI VDVVVMKDPK TKRSRGFGFI
TYSQSYMIDN AQNARPHKID GRTVEPKRAV PRQEIDSPNA GATVKKLFVG GLRDDHDEEC LREYFKDFGQ
IVSVNIVSDK DTGKKRGFAF IEFDDYDPVD KIILQKTHSI KNKTLDVKKA IAKQDMDRQG GGGGRGGPRA
GGRGGQGDRG QGGGGWGGQN RQNGGGNWGG AGGGGGFGNS GGNFGGQGG GSGGWNQQGG SGGGPWNNQG
GGNGGWNGGG GGGYGGGNSN GSWGGNGGGG GGGGGFGNEY QQSYGGGPQR NSNFGNNRPA PYSQGGGGGG
FNKGNQGGGQ GFAGNNYNTG GGGQGGNMGG GNRRY

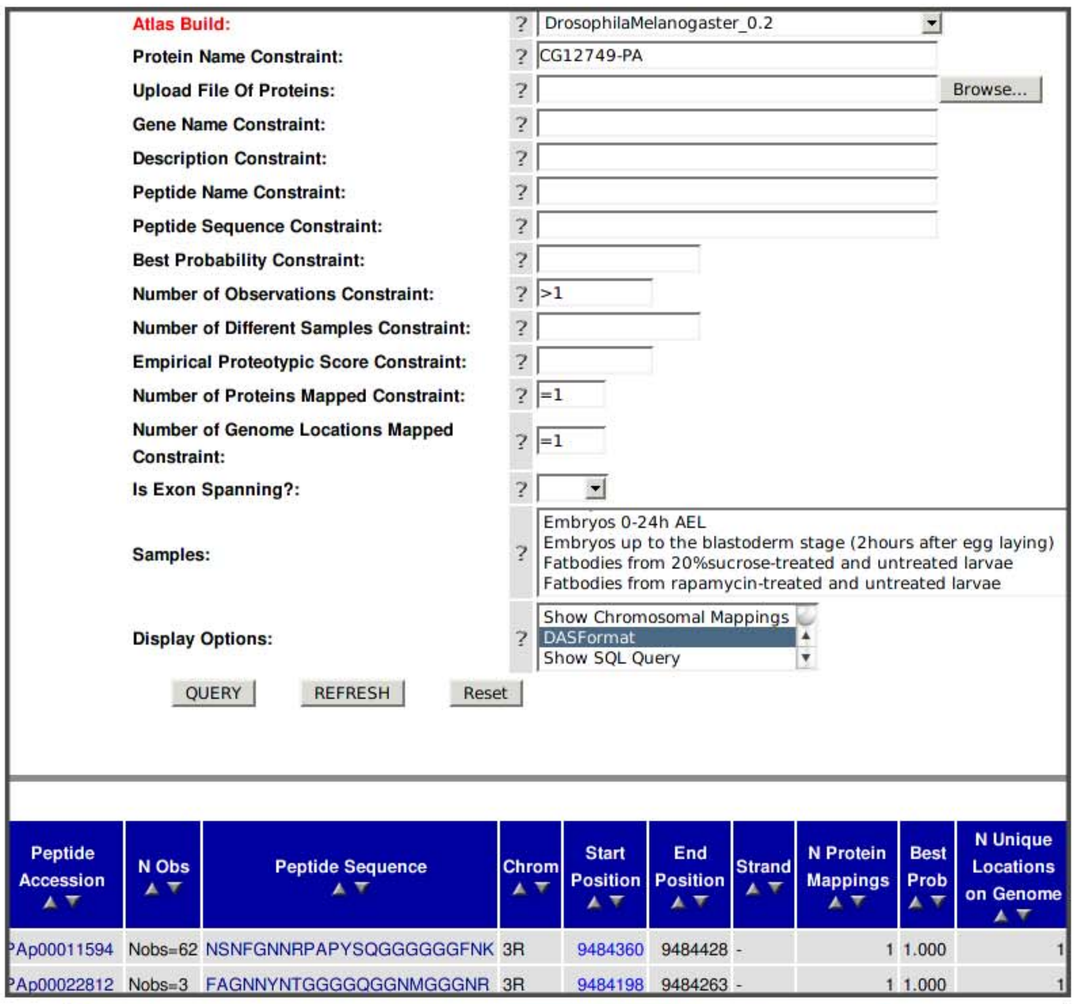
Atlas Build: DrosophilaMelanogaster_0.2
Protein Name Constraint: CG12749-PA
Upload File Of Proteins: Browse...
Gene Name Constraint:
Description Constraint:
Peptide Name Constraint:
Peptide Sequence Constraint:
Best Probability Constraint:
Number of Observations Constraint: >1
Number of Different Samples Constraint:
Empirical Proteotypic Score Constraint:
Number of Proteins Mapped Constraint: =1
Number of Genome Locations Mapped Constraint: =1
Is Exon Spanning?:
Samples:
Embryos 0-24h AEL
Embryos up to the blastoderm stage (2hours after egg laying)
Fatbodies from 20%sucrose-treated and untreated larvae
Fatbodies from rapamycin-treated and untreated larvae
Display Options:
Show Chromosomal Mappings
DASFormat
Show SQL Query
QUERY
REFRESH
Reset
Peptide Accession | N Obs | Peptide Sequence | Chrom | Start Position | End Position | Strand | N Protein Mappings | Best Prob | N Unique Locations on Genome
PAp00011594 | Nobs=62 | NSNFGNNRPAPYSQGGGGGGFNK | 3R | 9484360 | 9484428 | - | 1 | 1.000 | 1
PAp00022812 | Nobs=3 | FAGNNYNTGGGGQGGNMGGGNR | 3R | 9484198 | 9484263 | - | 1 | 1.000 | 1

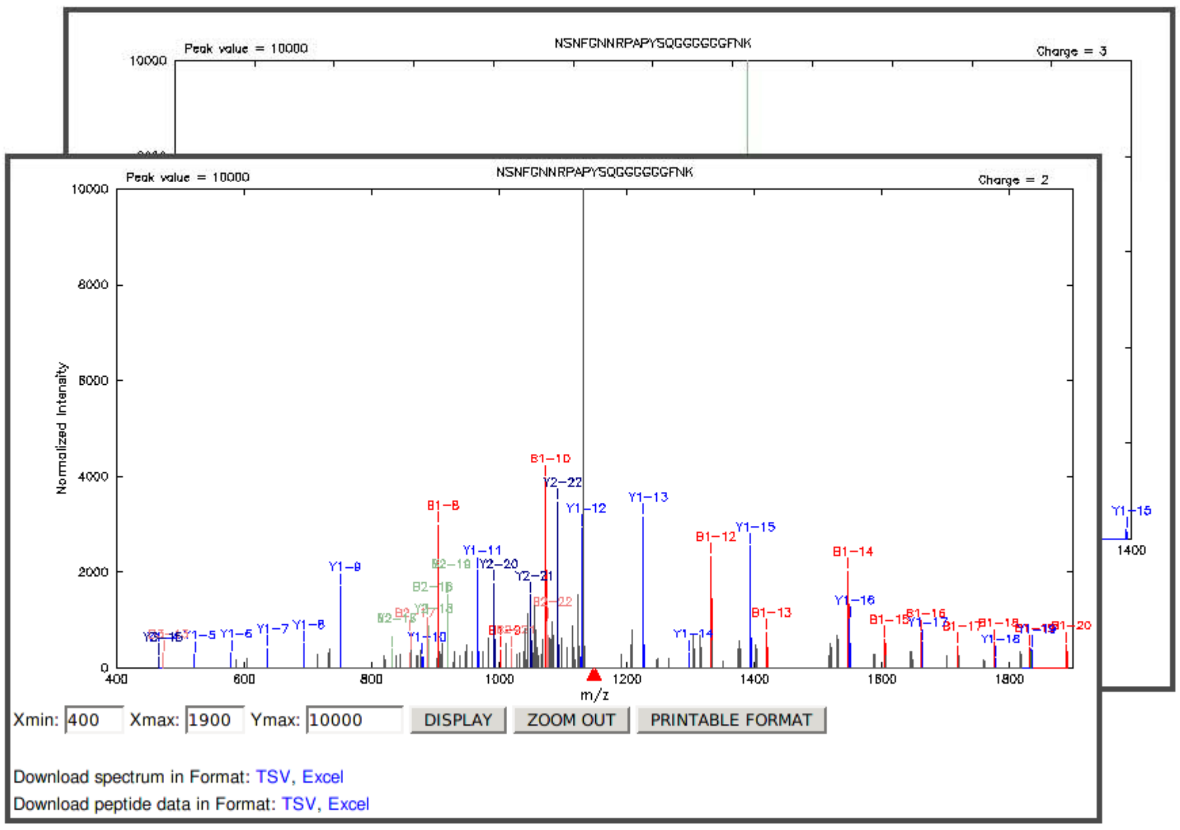
Peak value = 10000
NSNFGNNRPAPYSQGGGGGGFNK
Charge = 3
Peak value = 10000
NSNFGNNRPAPYSQGGGGGGFNK
Charge = 2
Normalized Intensity
m/z
Xmin: 400 Xmax: 1900 Ymax: 10000
DISPLAY
ZOOM OUT
PRINTABLE FORMAT
Download spectrum in Format: TSV, Excel
Download peptide data in Format: TSV, Excel

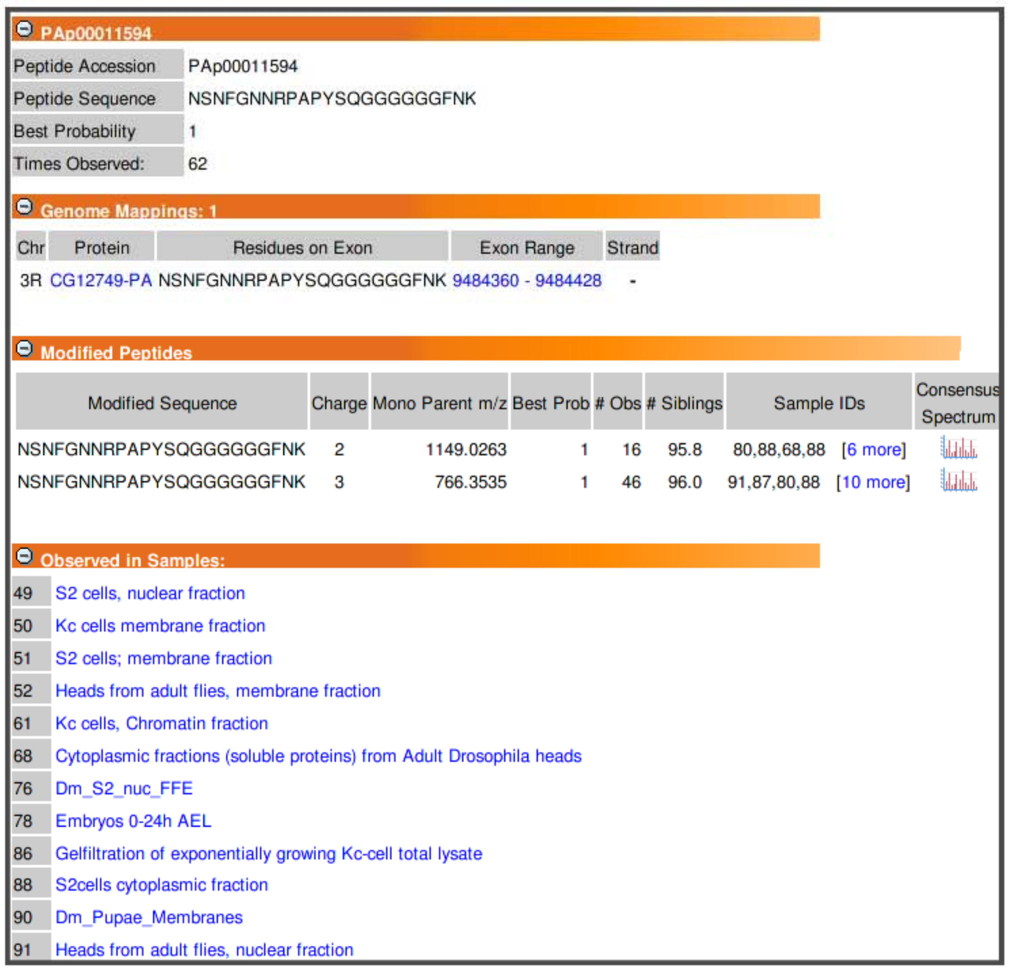
PAp00011594
Peptide Accession PAp00011594
Peptide Sequence NSNFGNNRPAPYSQGGGGGGFNK
Best Probability 1
Times Observed: 62
Genome Mappings: 1
Chr | Protein | Residues on Exon | Exon Range | Strand
3R | CG12749-PA | NSNFGNNRPAPYSQGGGGGGFNK | 9484360 - 9484428 | -
Modified Peptides
Modified Sequence | Charge | Mono Parent m/z | Best Prob | # Obs | # Siblings | Sample IDs | Consensus Spectrum
NSNFGNNRPAPYSQGGGGGGFNK | 2 | 1149.0263 | 1 | 16 | 95.8 | 80,88,68,88 [6 more]
NSNFGNNRPAPYSQGGGGGGFNK | 3 | 766.3535 | 1 | 46 | 96.0 | 91,87,80,88 [10 more]
Observed in Samples:
49 S2 cells, nuclear fraction
50 Kc cells membrane fraction
51 S2 cells; membrane fraction
52 Heads from adult flies, membrane fraction
61 Kc cells, Chromatin fraction
68 Cytoplasmic fractions (soluble proteins) from Adult Drosophila heads
76 Dm_S2_nuc_FFE
78 Embryos 0-24h AEL
86 Gelfiltration of exponentially growing Kc-cell total lysate
88 S2cells cytoplasmic fraction
90 Dm_Pupae_Membranes
91 Heads from adult flies, nuclear fraction
